# Strychnia in Intermittent Fever

**Published:** 1850-09

**Authors:** O. H. Edwards

**Affiliations:** Clinton, Summit County, Ohio


*Strychnia in Intermittent Fever.* By O. H. Edwards, M. D. of Clinton, Summit County, Ohio.

To the Editor of the Medical Examiner:

Dear Sir:—I noticed in your last number of the Medical Examiner, an article on the use of Strychnia in Intermittent Fever, by S. E. McKinly, M. D. of Williamsport, Tenn., wherein he says, "After having tested the various remedies used" in such cases, "none have proved so potent as Strychnos Nux Vomica;" thus assuming it to be of more value than Quinine, for which he offers it as a substitute. As my experience in the use of that article, which has been considerable both in intermittent fever, and other forms of disease, warrants no such conclusions, I offer you the history of a single case wherein I used it for a month without interruption.

Matthias Pool, aged 45 years; occupation farmer; has been subject to occasional attacks of circumorbital neuralgia for ten or twelve years. In the attacks which he had suffered for the last two years, I had been in the habit of bleeding him, and following it with mercurial cathartic and quinine, which always relieved him. As the attacks were becoming more frequent, I concluded to try the virtues of strychnia, in the next. Accordingly in the Spring of 1849, as he applied to me in an attack he was then laboring under, I bled him and gave him a mercurial cathartic, and put him on Tr. Strychnia, 40 minims* three times a day, to be gradually increased until the specific effects of the medicine should be experienced.

In consequence of his increasing the dose slowly, it was not until after he had used it four weeks, that he experienced the effects rising from the internal administration of full doses of that article. After it had occasioned nervous twitchings and involuntary muscular contractions, the dose was continued without being increased for three days—when having in a measure recovered from his neuralgia, it was thought best to interrupt its effects, and the strychnia was discontinued for a few days.

Upon the following day he had a regular shake of the ague—which was treated with quinine during the two subsequent inter-

*We presume Tr. Nucis Vomicæ is meant here, there being no officinal Tr. Strychniæ.—Ed.

missions, with the effect of removing the disease. I mention this case because of the notoriety that strychnia has of late obtained as a curative agent in the treatment of intermittent fever. Mr. Pool had not had intermittent fever for fifteen years preceding the attack to which I have referred, so that it was no case of relapse.

I had been using strychnia in a number of cases of intermittent fever previous to that time, both by itself and in conjunction with quinine, but never had been able to discover any virtues in it, and in the treatment of that disease, and after witnessing its effects in this particulur instance, I abandoned the hope of finding in it a subsitute for quinine.

